# Recurrence and patient reported outcomes after simultaneous bilateral versus unilateral groin hernia repair: prospective nationwide cohort study

**DOI:** 10.1093/bjsopen/zrag011

**Published:** 2026-03-11

**Authors:** Ramia Stolt, Hanna de la Croix, Henrik Holmberg, Maria Melkemichel, Agneta Montgomery, Benedit Witermark, Pär Nordin

**Affiliations:** Department of Surgery, Institute of Clinical Sciences, Sahlgrenska Academy, University of Gothenburg, Gothenburg, Sweden; Department of Surgery, Östersund Hospital, Östersund, Sweden; Department of Surgery, Institute of Clinical Sciences, Sahlgrenska Academy, University of Gothenburg, Gothenburg, Sweden; Department of Surgery, Sahlgrenska University Hospital, Region Västra Götaland, Gothenburg, Sweden; Department of Epidemiology and Global Health, Umeå University, Umeå, Sweden; Department of Clinical Science and Education, Söder Hospital, Karolinska Institute, Stockholm, Sweden; Department of Breast, Endocrine Tumors and Sarcoma, Karolinska University Hospital, Stockholm, Sweden; Department of Clinical Sciences, Malmö, Faculty of Medicine, Lund University, Lund, Sweden; Medical School Program, Umeå University, Umeå, Sweden; Department of Surgery, Östersund Hospital, Östersund, Sweden; Department of Diagnostics and Intervention, Umeå University, Umeå, Sweden

**Keywords:** pain, minimally invasive, laparoscopic, mesh, Sweden, asymptomatic

## Abstract

**Background:**

Bilateral groin hernias comprise approximately 25% of all groin hernias, with one side often being asymptomatic/minimally symptomatic. With an increase in minimally invasive approaches, simultaneous bilateral groin hernia repair (B-GHR) is frequently performed in routine practice. However, chronic pain and recurrence remain significant postoperative concerns. This study evaluated long-term outcomes after B-GHR *versus* unilateral groin hernia repairs (U-GHR).

**Methods:**

This nationwide population-based cohort study used prospective data from the Swedish Hernia Register, combined with a patient-reported outcome measure (PROM) questionnaire. All men and women aged ≥ 15 years with groin hernia repair registered between 1 September 2012 and 31 December 2018 were included in the study. Primary outcomes were chronic pain and patient dissatisfaction 1 year after B-GHR *versus* U-GHR. Secondary outcomes included reoperation for recurrence up until 2020, and risk factors for these long-term outcomes exclusively after B-GHR.

**Results:**

In all, 65 749 patients provided PROM data for analysis (response rate 69.4%). Chronic pain at 1 year was reported by 16.2% of patients (27) after B-GHR and by 15.4% of patients (9232) after U-GHR. A higher proportion of women undergoing B-GHR reported increased chronic pain than men (23.0 *versus* 15.4%; *P* < 0.001). Multivariable regression analyses revealed a higher risk of chronic pain (odds ratio (OR) 1.14; *P* = 0.002) and patient dissatisfaction (OR 1.30; *P* < 0.001) after B-GHR than U-GHR. Female sex and age < 50 years were independent risk factors for chronic pain and patient dissatisfaction after B-GHR. No significant difference was observed in reoperation rates for recurrence.

**Conclusions:**

B-GHR is associated with an increased risk of chronic pain and patient dissatisfaction compared with U-GHR. Women and younger patients are particularly at risk, suggesting a more cautious approach to simultaneous B-GHR in routine practice, especially in the absence of clear symptoms, and highlighting the importance of watchful waiting.

## Teaser

This large-scaled nationwide cohort study including both sexes found a significantly higher associated risk of both chronic pain and patient dissatisfaction 1 year after bilateral compared with unilateral groin hernia repair. Women and younger patients are particularly at risk, suggesting a more cautious approach to simultaneous bilateral groin hernia repair in routine practice, especially in the absence of clear symptoms, and highlighting the importance of watchful waiting.

## Introduction

Groin hernia repair (GHR) is one of the most frequently performed surgical procedures worldwide^1,2^. Bilateral groin hernias account for up to 25% of all groin hernias, with an estimated prevalence of asymptomatic contralateral groin hernias of nearly 13%^1,2^. Minimizing long-term complications following GHR, such as chronic pain and recurrence, is crucial for optimizing patient outcomes and quality of life^1,3^. Assessments of chronic pain rates following GHR vary widely across the literature due to inconsistent definitions being used^1,4,5^. Current guidelines estimate that 10–12% of patients experience moderate to severe chronic pain ≥ 3 months after GHR^3^. However, recent register-based studies report significantly higher chronic pain rates 1 year after surgery, particularly in women (18.4%) and men aged < 50 years (19.4%)^6,7^.

Approximately one-third of primary groin hernias are asymptomatic or minimally symptomatic at diagnosis, and nearly 75% of these will eventually become symptomatic, necessitating surgery at a later stage^1,8^. Although watchful waiting has been established as a safe management for men in terms of mortality rates and risk of emergency repair, evidence supporting this strategy in women is lacking^9,10^. Historically, prophylactic surgery was often recommended to potentially prevent acute strangulation and its associated higher mortality^11^. However, this risk is almost exclusively linked to female hernias and incarcerated femoral hernias, which often present without previous symptoms and are therefore not always preventable, even with a rising number of elective repairs^11,12^.

The international HerniaSurge guidelines^1^ recommend laparoscopic repair for bilateral groin hernias, provided the approach is clinically feasible and consistent with patient preferences. Nonetheless, there is conflicting published data regarding simultaneous treatment of bilateral groin hernias^13,14^. A recent systematic review^14^ concluded that simultaneous bilateral repairs, despite one side being asymptomatic, offered more benefits than risks for both the patient and society. However, other studies,^15^ including the updated international guidelines,^16^ suggest a more cautious surgical approach. In addition, most research on postoperative chronic pain primarily focuses on individual risk factors and different surgical methods^17–20^. The evidence in the literature supporting prophylactic repairs for asymptomatic/minimally symptomatic contralateral groin hernias is scarce and the long-term outcomes of simultaneous bilateral groin hernia repair (B-GHR) remain unexplored, particularly in women^13,21,22^.

Given the long-term complications, does prophylactic B-GHR for a contralateral groin hernia cause more harm than benefits for the patient? This study, with data from the Swedish Hernia Register including both men and women, aimed to assess chronic pain, patient dissatisfaction, and reoperation for recurrence following simultaneous bilateral repair, with patients undergoing unilateral groin hernia repair (U-GHR) serving as the control group.

## Methods

### Study design

This nationwide observational cohort study used prospectively recorded data from the Swedish Hernia Register (SHR), combined with patient-reported outcome measures (PROM) questionnaire integrated in the registry, and adhered to the STROBE guidelines^23^ (*Appendix S1*). Data derived from the register were handled in a cumulative manner on a national level and followed the Declaration of Helsinki. Ethics approval for the study protocol was obtained from the Swedish Ethical Review Authority (Dnr 2023-01969-01) before data extraction.

### Study population

**Fig. 1:**
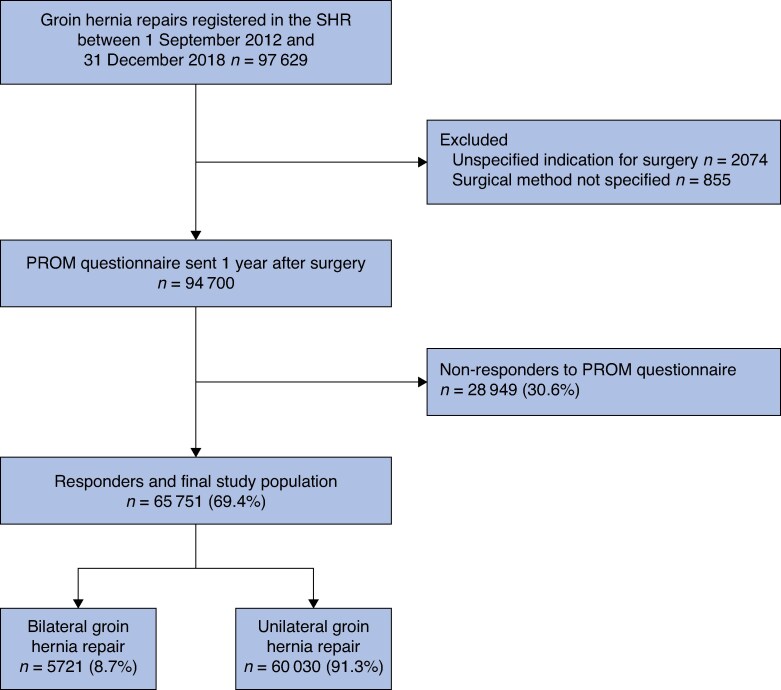
Flow chart of the included study population SHR, Swedish Hernia Register; PROM, patient-reported outcome measure.

All men and women aged ≥ 15 years with a groin hernia repair recorded in the SHR between 1 September 2012 and 31 December 2018 were eligible for inclusion in the study (*Fig. 1*). The included hernia repairs comprised both open repairs (open anterior mesh repair, open posterior mesh repair, combined anterior and posterior mesh repairs, repairs with plugs, and suture repairs) and laparoscopic repairs (totally extraperitoneal (TEP) and transabdominal preperitoneal (TAPP)). Despite a preference for laparoscopic repair for bilateral hernias, open bilateral repair is still performed in Sweden, particularly for older patients or those with American Society of Anesthesiologists (ASA) grade III–IV, justifying the inclusion of both approaches. The inclusion criteria were regardless of the mode of admission (elective *versus* emergency repair) or whether the repair was for primary or recurrent hernia. Exclusions were limited to repairs performed due to chronic pain after previous repair, mesh infections, or registrations with incomplete data on surgical method (*Fig. 1*). Only patients responding to the PROM questionnaire (responders) were included in the subsequent statistical analysis (*Fig. 1* and *Table 1*).

**Table 1:** Baseline patient and procedural characteristics

	B-GHR (*n* = 5721)	U-GHR (*n* = 60 028)	P*	Total repairs (*n* = 65 749)
**Sex**				
Male	5130 (89.7%)	54 536 (90.9%)	0.003	59 666 (90.7%)
Female	591 (10.3%)	5492 (9.1%)		6083 (9.3%)
**Age (years)**				
< 50	990 (17.3.%)	9685 (16.1%)	< 0.001	10 675 (16.2%)
50–70	3042 (53.2%)	28 046 (46.7%)		31 088 (47.3%)
> 70	1689 (29.5%)	22 297 (37.1%)		23 986 (36.5%)
Median (range)	64.2 (15.7–93.5)	66.4 (15.3–102)		66.2 (15.3–102)
**ASA grade**†				
I–II	5189 (90.7%)	52 604 (87.6%)	< 0.001	57 793 (87.9%)
III	531 (9.3%)	7419 (12.4%)		7950 (12.1%)
IV–V	1 (0.0%)	5 (0.0%)		6 (0%)
**Surgical technique**				
Open repair	732 (12.8%)	49 829 (83.0%)	< 0.001	50 561 (76.9%)
Endolaparoscopic repair	4989 (87.2%)	10 199 (17.0%)		15 188 (23.1%)
**Hernia anatomy**				
Lateral	2469 (43.2%)	34 628 (57.7%)	< 0.001	37 097 (56.4%)
Medial	2509 (43.9%)	18 940 (31.6%)		21 449 (32.6%)
Femoral	248 (4.3%)	1868 (3.1%)		2116 (3.2%)
Combined‡	495 (8.7%)	4592 (7.6%)		5087 (7.7%)
**Patient satisfaction**				
Score 1–2: Satisfied	5310 (92.8%)	57 012 (95.0%)	< 0.001	62 322 (94.8%)
Score 3–4: Dissatisfied	411 (7.2%)	3016 (5.0%)		3427 (5.2%)
**Chronic pain (IPQ)**				
Score 1–3: No pain	4794 (83.8%)	50 796 (84.6%)	0.099	55 590 (84.5%)
Score 4–7: Pain	927 (16.2%)	9232 (15.4%)		10 159 (15.5%)
**Proportion of patients with pain (IPQ score 4–7)**				
Male	791 (15.4%)	8243 (15.1%)	< 0.001	9034 (15.1%)
Female	136 (23.0%)	989 (18.0%)		1125 (18.5%)
**Reoperation for recurrence**				
Yes	202 (3.5%)	1330 (2.2%)	< 0.001	1532 (2.3%)
No	5519 (96.5%)	58 698 (97.8%)		64 217 (97.7%)
**Follow-up time (years)§**				
Mean(s.d.)	4.75(1.86)	4.92(1.86)		4.90(1.86)
Median (range)	4.68 (0–8.18)	4.82 (0–8.18)		4.80 (0–8.18)

Values are *n* (%) unless otherwise stated. †Physical status classification system adopted in 1963 by the ASA. Grade I–II refers to people who are healthy or have a mild systemic disease; grade III–IV refers to people who have severe systemic diseases, some of which are life threatening. ‡Combined hernias are defined as lateral hernias combined with either cord lipoma or medial hernia. Any combination with a femoral hernia is defined primarily as a femoral hernia in the Swedish Hernia Register. §Follow-up time for reoperation due to recurrence up to 6 November 2020. B-GHR, bilateral groin hernia repair; U-GHR, unilateral groin hernia repair; ASA, American Society of Anesthesiologists; IPQ, Inguinal Pain Questionnaire. *A χ^2^ cross-tabulation was performed for each categorical variable in the bilateral and unilateral cohorts, with *P* values as given.

### Swedish Hernia Register

The SHR, established in 1992 with achievement of national data completeness by 2004, captures approximately 97% of all GHRs performed in Sweden on patients aged ≥ 15 years^24^. To date, the SHR includes data from over 400 000 prospectively registered GHRs^25^. Each patient and surgery are tracked using the patient’s unique personal identity number, allowing for accurate data on death or reoperations for recurrence, regardless of where in the country the reoperation occurs^26^. The SHR collects detailed information on patient characteristics, surgical method, type of anaesthesia, hernia anatomy, and materials used. All variables are entered online by the operating surgeon at the time of surgery, and annual validation of participating units ensures the accuracy and completeness of the data^24,25^.

### PROM questionnaire

Between 2012 and 2018, the SHR routinely sent a PROM questionnaire to all patients 1 year after surgery to assess chronic pain and patient satisfaction. An additional reminder was sent out after 30 days.

#### Chronic pain

The first question in the PROM questionnaire was item 2 from the highly validated Inguinal Pain Questionnaire (IPQ)^27^. Patients were asked to grade their worst pain in the operated groin over the past week using a seven-point scale^28^. The scores were defined as following: 1, no pain; 2, pain present, but easily ignored; 3, pain present, cannot be ignored, but does not interfere with everyday activities; 4, pain present, cannot be ignored, and interferes with concentration on everyday activities; 5, pain present, interferes with most activities; 6, pain present, necessitating bed rest; and 7, pain present, prompt medical advice sought. Scores of 1–3 were defined as no pain, whereas scores of 4–7 were categorized as pain, consistent with the HerniaSurge Group’s guideline definition of chronic pain^1^. The reliability of these short-form questionnaires has been validated as equivalent to regular IPQs^28,29^.

#### Patient dissatisfaction

The second question in the PROM questionnaire evaluated patient satisfaction with their GHR, with possible answers of: 1, yes, satisfied; 2, yes, almost satisfied; 3, no, mainly not satisfied; and 4, no, not at all satisfied. Patient satisfaction was defined as scores 1–2, whereas patient dissatisfaction was defined as scores 3–4.

### Bilateral repair

Bilateral repair involved either an open or laparoscopic technique at a single surgical admission. In the SHR, bilateral operations are registered as two separate entries (that is, one for each side) and are linked for analysis. However, the SHR lacks data regarding the symptomatology or indication for B-GHR. Between 2012 and 2018, these patients received only one PROM questionnaire despite undergoing a bilateral repair, capturing patient-reported outcome measures (that is, chronic pain and dissatisfaction) from one single site. In assessment of reoperation rates for recurrence, a single surgical admission for bilateral repair was analysed as a single unit, rather than assessing each individual repair separately.

### Study objectives

The primary outcomes were chronic pain and patient dissatisfaction 1 year after B-GHR compared with U-GHR.

Secondary outcomes involved reoperation for recurrence following B-GHR *versus* U-GHR and identifying risk factors for chronic pain, patient dissatisfaction, and reoperation for recurrence exclusively after B-GHR. Reoperation for recurrence was defined as a subsequent hernia repair in the same groin as the index surgery registered in the SHR, regardless of the laterality, with only one reoperation for recurrence in each groin being considered. Follow-up time for reoperation events continued until 6 November 2020.

### Statistical analysis

Descriptive statistics for B-GHR and U-GHR are presented in *Table 1*, with categorical variables described as numbers and percentages. Age is reported as a median and was further divided into three subgroups. A χ^2^ test was used to compare categorical variables, with *P* values reported. A reciprocal Kaplan–Meier plot was used to illustrate the cumulative reoperation rate for recurrence after B-GHR and U-GHR Multivariable logistic regression analysis was used to assess the risks of chronic pain and patient dissatisfaction in the PROM-responding population after B-GHR *versus* U-GHR, whereas Cox proportional analyses were used to estimate the risk of reoperation for recurrence (*Table 2*). Participants were censored at the time of death, emigration, or upon reaching the end of the study period. Additional sex-stratified analyses were performed to separately investigate these outcomes in men and women (*Table 2*). Similar analyses were used for the outcomes exclusively after B-GHR, aiming to identify associated risk factors (*Table 3*). All analyses were adjusted for age, hernia anatomy, and surgical technique based on their significant impact on the exposure and associated surgical outcome. Odds ratios (OR) and hazards ratios (HR) were estimated, along with their 95% confidence interval (c.i.). Statistical significance was set at *P* < 0.05. All statistical analyses were conducted using SPSS^®^ Statistics version 29.0.2.0 (IBM, Armonk, NY, USA).

**Table 2:** Multivariable regression analyses for the risk of chronic pain, patient dissatisfaction, and reoperation for recurrence following B-GHR

	U-GHR	B-GHR	*P*
**Outcomes for total repairs**			
Chronic pain*	1.0	aOR 1.14 (1.05, 1.24)	0.002
Patient dissatisfaction*	1.0	aOR 1.30 (1.15, 1.47)	< 0.001
Reoperation for recurrence†	1.0	aHR 1.13 (0.95, 1.35)	0.175
**Outcomes for male repairs**			
Chronic pain‡	1.0	aOR 1.08 (0.98, 1.19)	0.111
Patient dissatisfaction‡	1.0	aOR 1.19 (1.04, 1.37)	0.012
Reoperation for recurrence§	1.0	aHR 0.99 (0.82, 1.18)	0.879
**Outcomes for female repairs**			
Chronic pain‡	1.0	aOR 1.47 (1.19, 1.81)	< 0.001
Patient dissatisfaction‡	1.0	aOR 1.36 (0.98, 1.89)	0.070
Reoperation for recurrence§	1.0	aHR 1.91 (1.02, 3.57)	0.042

Values in parentheses are 95% confidence intervals. *Multivariable logistic regression analysis and †multivariable Cox proportional analysis adjusted for age (< 50, 50–70, > 70 years; age < 50 years as the reference), sex (male as the reference), surgical method (open repair as the reference), and hernia anatomy (lateral hernia as the reference). ‡Multivariable logistic regression analysis and §multivariable Cox proportional analysis adjusted for age (< 50, 50–70, > 70 years; age < 50 years as the reference), surgical method (open repair as the reference), and hernia anatomy (lateral hernia as the reference). B-GHR, bilateral groin hernia repair; U-GHR, unilateral groin hernia repair; aOR, adjusted odds ratio; aHR, adjusted hazard ratio.

**Table 3:** Multivariable logistic and Cox proportional regression analyses for the outcomes exclusively after bilateral groin hernia repair

	Chronic pain*		Patient dissatisfaction*		Reoperation for recurrence†	
	aOR	*P*	aOR	*P*	aHR	*P*
**Sex**						
Male (Reference)	1.0		1.0		1.0	
Female	1.48 (1.19, 1.85)	<0.001	1.02 (0.72, 1.44)	0.911	0.56 (0.31, 1.03)	0.064
**Age (years)**						
> 70 (Reference)	1.0		1.0		1.0	
50–70	0.97 (0.82, 1.15)	0.721	1.56 (1.2, 2.03)	<0.001	0.99 (0.72, 1.35)	0.931
< 50	1.36 (1.11, 1.86)	<0.001	2.47 (1.83, 3.34)	<0.001	0.69 (0.44, 1.1)	0.122
**Surgical technique**						
Open repair (Reference)	1.0		1.0		1.0	
Laparoscopic repair	0.77 (0.63, 0.95)	0.014	0.60 (0.45, 0.78)	<0.001	1.09 (0.71, 1.66)	0.694
**Hernia anatomy**						
Lateral (Reference)	1.0		1.0		1.0	
Medial	0.83 (0.71, 0.97)	0.016	0.95 (0.76, 1.18)	0.635	0.94 (0.70, 1.26)	0.660
Femoral	1.19 (0.85, 1.66)	0.303	1.29 (0.79, 2.09)	0.309	0.78 (0.34, 1.83)	0.573

Values in parentheses are 95% confidence intervals. *Multivariable logistic regression analysis. †Multivariable Cox proportional analysis. aOR, adjusted odds ratio; aHR, adjusted hazard ratio.

## Results

In all, 97 629 GHRs were registered in the SHR during the study period. Of the patients who met the inclusion criteria, 65 749 responded to the PROM questionnaire (response rate 69.4%) and were included in the study population for statistical analysis (*Fig. 1*).

### Patient characteristics

Simultaneous B-GHR accounted for 8.7% (5721) of all included repairs (*Table 1*). The B-GHR group had a higher proportion of women than the U-GHR group (10.3 *versus* 9.1%, respectively; *Table 1*). The median age of B-GHR and U-GHR groups was 64.2 and 66.4 years, respectively (*Table 1*). B-GHR was predominantly performed using laparoscopic techniques (87.2%), compared with only 17% of U-GHR performed using laparoscopic techniques. More medial and femoral hernias were found in the B-GHR group than in the U-GHR group (*Table 1*).

### Chronic pain

The overall chronic pain rate 1 year after B-GHR was 16.2%, compared with 15.4% after U-GHR (*Table 1*). Women undergoing B-GHR had a higher rate of chronic pain than men (23.0 *versus* 15.4%, respectively; *Table 1*). Multivariable regression analysis revealed a significantly increased risk of chronic pain following B-GHR than U-GHR (OR 1.14; 95% c.i. 1.05 to 1.24; *P* = 0.002; *Table 2*). When stratified by sex, women undergoing B-GHR had a higher risk of chronic pain than women undergoing U-GHR (OR 1.47; 95% c.i. 1.19 to 1.81; *P ≤* 0.001; *Table 2*). This was not seen in men.

### Patient dissatisfaction

Patient dissatisfaction 1 year after surgery was 7.2% following B-GHR and 5.0% after U-GHR (*Table 1*). Dissatisfied patients reported chronic pain more frequently after B-GHR than U-GHR (44.3 *versus* 32.7%, respectively; *P ≤* 0.001). Similarly, 26.2% of those reporting chronic pain after B-GHR were dissatisfied with their surgery, compared with 19.3% after U-GHR (*P ≤* 0.001). Multivariable regression analysis revealed a significantly increased OR for patient dissatisfaction after B-GHR (1.30; 95% c.i. 1.15 to 1.47; *P ≤* 0.001; *Table 2*). In sex-stratified analysis, men undergoing B-GHR had an elevated risk of patient dissatisfaction than men undergoing U-GHR (OR 1.19; 95% c.i. 1.04 to 1.37; *P* = 0.012), which was not seen in women (*Table 2*).

### Reoperation for recurrence

**Fig. 2:**
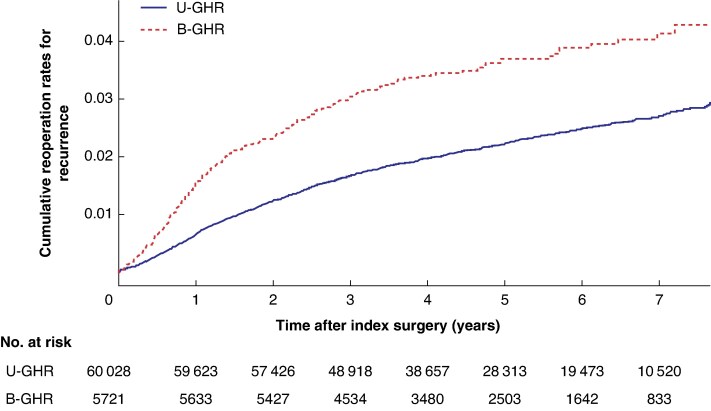
Reciprocal Kaplan–Meier plot showing cumulative reoperation rates for recurrence following U-GHR and B-GHR U-GHR, unilateral groin hernia repair; B-GHR, bilateral groin hernia repair.

The cumulative reoperation rate for recurrence among PROM responders is shown in *Fig. 2*. The observed reoperation rate for recurrence was 3.5% after B-GHR and 2.2% after U-GHR (*Table 1*). Multivariable Cox proportional analysis revealed a non-significant increase in the HR after B-GHR (1.13; 95% c.i. 0.95 to 1.35; *P* = 0.175; *Table 2*). Sex-stratified analysis showed a significantly higher risk of reoperation for recurrence in women undergoing B-GHR compared with those undergoing U-GHR (HR 1.91; 95% c.i. 1.02 to 3.57; *P* = 0.042; *Table 2*). This was not seen for men. An additional multivariable Cox regression analysis for reoperation due to recurrence including both responders and non-responders to the PROM questionnaire (data not shown) demonstrated a similar non-significant estimate for B-GHR *versus* U-GHR with an HR of 1.14 (95% c.i. 0.99 to 1.32; *P* = 0.064).

### Risk factors for outcomes exclusively after B-GHR

Women undergoing B-GHR had a significantly higher risk of chronic pain compared with men (OR 1.48; 95% c.i. 1.19 to 1.85; *P ≤* 0.001; *Table 3*). Age < 50 years was also associated with a significantly elevated risk of both chronic pain (OR 1.36; 95% c.i. 1.11 to 1.86; *P ≤* 0.001) and patient dissatisfaction (OR 2.47; 95% c.i. 1.83 to 3.34; *P ≤* 0.001) 1 year after surgery (*Table 3*). No statistically significant risk factors were identified for reoperation for recurrence.

### Non-responders to the PROM questionnaire

The baseline characteristics of the 28 949 GHRs in patients who did not respond to the PROM questionnaire were similar to the group of responders responding group (*Supplementary Table S1*). Simultaneous B-GHR accounted for 8.2% of the repairs, with 10.4% of them performed on women. The median age of PROM non-responders was lower (57.7 and 61.1 years for the B-GHR and U-GHR cohorts, respectively) than that of the PROM responders. Among non-responders, 12% in the B-GHR group and 16.2% in the U-GHR group were classified as ASA grade ≥ III (*versus* 9.5 and 12.4%, respectively, in the PROM responder cohort). Among non-responders, the reoperation rate for recurrence was 4.5% in the B-GHR group and 2.6% in the U-GHR (*versus* 3.5 and 2.2%, respectively, in the PROM responder cohort).

## Discussion

This nationwide population-based register study provides a comprehensive analysis of long-term outcomes following simultaneous B-GHR *versus* U-GHR in both men and women. Key findings indicate significantly higher chronic pain (rates up to 23% in women and 15% in men) and patient dissatisfaction 1 year after bilateral repairs, but with comparable reoperation rates for recurrence. Specifically, women and younger patients have an associated increased risk of developing chronic pain after B-GHR.

The conflicting published data on the management of bilateral groin hernias with one side being asymptomatic or occult raise concerns. Gass *et al*.^30^ concluded that simultaneous bilateral repair is a highly effective therapeutic approach. However, that study only assessed the immediate postoperative complications (for example, wound infection, haematoma, seroma, pneumonia, or urinary tract infection) when comparing 3048 bilateral TEP repairs to 3457 unilateral TEP repairs between 1995 and 2006^30^. Only 3.7% of the patients in that study^30^ were women and no long-term outcomes, such as chronic pain or reoperation for recurrence, were investigated. Similarly, Köckerling *et al*.^15^ analysed 2695 bilateral TEP repairs in a predominantly male population (94.6%), and found a significantly higher incidence of intraoperative bladder injuries after bilateral TEP repairs compared with 6700 unilateral TEP repairs. Without accounting for long-term complications, Köckerling *et al*.^15^ opposed the recommendation for prophylactic B-GHR. Meanwhile, in their meta-analysis incorporating data from 1774 patients across six non-randomized studies, Park *et al*.^14^ underscored the potential benefits of prophylactic bilateral repairs (for example, prevention of a subsequent operation later in life, reduced peri- and early postoperative complications, shorter hospitalization, and early return to daily activities). Conversely, other studies^4,13,15,17^ suggest a more cautious approach for bilateral groin hernias considering postoperative complications and the potential risk of chronic pain.

The present study demonstrates significantly higher chronic pain rates, particularly in women, at the 1-year follow-up after B-GHR compared with U-GHR, exceeding previously reported estimates^1,3^. Notably, these elevated rates may be underestimated, because patients with bilateral repair received only one PROM questionnaire capturing complaints from a single groin, despite both sides being repaired. As demonstrated by multivariable regression analyses, B-GHR independently increased the risk of both chronic pain and patient dissatisfaction 1 year after surgery compared with U-GHR. The laparoscopic approach was protective against both these outcomes, supporting the acknowledged benefits of minimally invasive procedures^4,16,31^. However, although women predominantly had laparoscopic repairs, they still exhibited a significantly higher risk of developing chronic pain following B-GHR, both in comparison to men and in sex-stratified analyses. This persistent disparity suggests that anatomical and sex-specific factors may influence postoperative pain and surgical outcomes in women^7,32^. Dissatisfied patients reported chronic pain more often after bilateral than unilateral repair, and equally, those with chronic pain were more often dissatisfied.

Younger age (< 50 years) independently increased the risk of both chronic pain and patient dissatisfaction after B-GHR, confirming the established association between younger age and chronic pain. Conversely, older individuals may be less likely to report complaints, potentially reflecting greater postsurgical resilience or altered pain perception with advancing age.

Simultaneous B-GHR did not increase the risk of reoperation for recurrence compared with U-GHR among PROM responders. There was no statistically significant difference between the B-GHR and U-GHR groups when the PROM non-responding population was included in the analysis. The present study did not confirm the previously expressed^4,21^ concerns of higher reoperation rates for recurrence initially observed after laparoscopic unilateral repairs. However, women undergoing B-GHR had a higher risk of reoperation for recurrence than women undergoing U-GHR, despite the majority in both groups undergoing laparoscopic repair.

Watchful waiting is a safe management strategy for men with bilateral hernias; however, its applicability and safety in women require further investigation, ideally through randomized clinical trials.

The main strength of the present study lies in its extensive data set, comprising a large-scaled cohort of B-GHR conducted in modern times and combined with a nearly 70% response rate to the PROM questionnaire. The inclusion of nationwide unselected GHRs within the SHR provides substantial power to detect rare associations influencing patient and surgical outcomes. The high number of included bilateral repairs, particularly in women, and the use of both open and laparoscopic techniques make this a unique and highly comprehensive study. Despite a preference for laparoscopic repair for bilateral hernias, open bilateral repair is still performed in Sweden, particularly for older patients or those with ASA grade III–IV, justifying the inclusion of both surgical approaches. The findings of this study challenge previous assumptions of prophylactic B-GHR, because most of the reported studies were conducted mainly on men with insufficient data on long-term complications. Population-based nationwide registry data are more likely to provide real-world results than data from specialized high-volume centres. The annual validation of the SHR further reduces the risk of bias. The availability of data on PROM non-responders allows comparison to the responding cohort. Although PROM collection was temporally paused between 2018 and 2023 due to the COVID-19 pandemic and subsequent registry restructuring, the study period extending to 2020 ensured a minimum 2-year follow-up for reoperation due to recurrence. In addition, the preferential use of TEP over TAPP repairs in Sweden reduces the likelihood of potentially finding and overtreating occult contralateral groin hernias, as may occur with TAPP repairs^1^.

The primary limitation of present study is that the 5721 bilateral repairs among responders accounted for only 8.7% of all registered GHRs in the SHR. The comparison was made against a larger group undergoing unilateral repair rather than between one-session bilateral repairs *versus* two-session bilateral repairs (that is, two unilateral repairs performed sequentially). Due to the limited number of two-session bilateral repairs, this analysis was not feasible and remains an area for future exploration. The disparity between the lower observed prevalence of bilateral hernias in this study compared with the numbers reported in the literature likely reflects Sweden’s conservative approach to operating mainly on symptomatic and clinically confirmed bilateral hernias, particularly in patients with ASA grade III–V. The reported rate of 8.7% bilateral repairs reflects the proportion of B-GHRs performed in the country, not the true prevalence of bilateral hernias in the population, which remains difficult to estimate due to limited reliable data in the literature. In addition, the predominant use of TEP repair in Sweden may contribute to potential underdiagnosis of metachronous bilateral hernias, because this approach offers limited visualization of the contralateral side.

A simple assumption may suggest that B-GHR would result in twice the risk of chronic pain or reoperation for recurrence compared with U-GHR. However, this could not be confirmed in the present study. The reoperation rates for recurrence were comparable between the B-GHR and U-GHR groups, whereas pain assessment only involved one groin site, even in cases of bilateral repair. Although the difference in chronic pain rates between U-GHR and B-GHR is statistically significant with increased risk, the absolute risk difference can be considered small. However, it may still have important clinical relevance when considered in the broader context of patient outcomes. Even small reductions in chronic pain can yield meaningful improvements in quality of life, particularly at the population level. Hence, continued efforts to reduce chronic pain outcomes is of utmost importance, further highlighting the need to study this relatively low-volume group of groin hernias using large-scale cohorts.

The PROM questionnaire distributed 1 year after surgery to each patient rather than for each repair performed may address another notable limitation of the study, potentially underestimating chronic pain and patient dissatisfaction after bilateral repairs. Notably, the relatively high response rate may suggest a potential overestimation of these outcomes among responders. However, non-responders had a significantly lower median age than the responding population, and in previous register-based studies non-responders were confirmed to have less chronic pain^4^. The patient dissatisfaction designed to capture aspects of patient reported experience measures, such as communication and perceived quality of care, has been used in previously published studies through the SHR^4^. However, this item has not undergone formal validation, which may limit its interpretative value and reliability. The reasons for the observed sex-based disparity in patient dissatisfaction remain unclear, and are likely multifactorial (for example, communication, waiting time, postoperative discomfort), and hence beyond the scope of the present study. Finally, this study can only conclude the reoperation rate for recurrence, and not the actual recurrence rates, which may, in reality, be as high as 40%^33^.

## Supplementary Material

zrag011_Supplementary_Data

## Data Availability

All data are uncoded and can be provided on request.
