# Supplementary material for: Recurrence and patient reported outcomes after simultaneous bilateral versus unilateral groin hernia repair: prospective nationwide cohort study
Source: BJS Open. 2026 Mar 11;10(2):zrag011. doi: 10.1093/bjsopen/zrag011 (PMC12980330; doi:10.1093/bjsopen/zrag011)
Supplement: zrag011_Supplementary_Data [file zrag011_supplementary_data.docx]

**Title**

“Increased Chronic pain and Patient Dissatisfaction after simultaneous Bilateral groin hernia repair: *prospective nationwide cohort study”*

**Authors**

# Ramia Stolt^1,2^, Hanna de la Croix^1,3^, Henrik Holmberg^4^, Maria Melkemichel^5,6^, Agneta Montgomery^7^, Benedit Witermark^8^ and Pär Nordin^2,9^.

**Affiliations:** Institution/Departments

^1^Department of Surgery, Institute of Clinical Sciences, Sahlgrenska Academy, University of Gothenburg, Sweden.

^2^Department of Surgery, Östersund Hospital, Östersund, Sweden.

^3^Department of Surgery, Sahlgrenska University Hospital, Region Västra Götaland, Gothenburg, Sweden.

^4^Department of Epidemiology and Global Health, Umeå University, Umeå, Sweden.

^5^Department of Clinical Science and Education, Söder Hospital, Karolinska Institute, Stockholm, Sweden.

^6^Department of Breast, Endocrine Tumors and Sarcoma, Karolinska University Hospital, Stockholm, Sweden.

^7^Department of Clinical Sciences, Malmö, Faculty of Medicine, Lund University, Lund, Sweden.

^8^Medical School Program, Umeå University, Umeå, Sweden.

^9^Department of Diagnostics and Intervention, Umeå University, Umeå, Sweden.

**Corresponding author.**

Ramia Stolt

Östersund Hospital, Department of Surgery, Kyrkgatan 16, 831 83 Östersund, Sweden.

Email address: [ramia.stolt@gu.se](mailto:ramia.stolt@gu.se)

ORCID iD: 0009-0002-0202-464X

**Supplementary Materials - Index**

| **Supplementary Results** |  |
| --- | --- |
| **Table S1:** Baseline patient and procedural characteristics for non-responders. | ***pag.* 9** in the main Manuscript. |
| **Supplementary Figures and Tables** |  |
| **Table S2:** Unadjusted analysis for the risk of chronic pain, patient dissatisfaction and reoperation for recurrence for the included study population. | ***pag.* 9** in Response to Reviewers. |

**Supplementary Appendixes *pag.* 5** in the main Manuscript.

**STROBE** Statement - checklist of items that should be included

in reports of observational studies.

**Table S1:** Baseline patient and procedural characteristics for non-responders.

|  | B-GHR  (n=2 393) | U-GHR  (n= 26 556) | *p*-value^c^ |
| --- | --- | --- | --- |
| Sex  Men  Women | 2 143 (89.6)  250 (10.4) | 23 603 (88.9)  2 953 (11.1) | 0.315 |
| Age (years)  < 50  50 – 70  > 70  Median [min, max] | 752 (31.3)  1 115 (46.6)  526 (22.0)  57.7 [17.2,102] | 8 061 (30.4)  10 102 (38.0)  8 393 (31.6)  61.0 [15.0,102] | <0.001 |
| ASA fitness grade^a^  I-II  III  IV-V | 2 107 (88.0)  286 (12.0)  0 (0.0) | 22 247 (83.8)  4 302 (16.2)  7 (0.0) | <0.001 |
| Surgical technique  Open repair  Endo-laparoscopic repair | 413 (17.3)  1 980 (82.7) | 21 290 (80.2)  5 266 (19.8) | <0.001 |
| Hernia anatomy  Lateral  Medial  Femoral  Combined^b^ | 1 078 (45.0)  989 (41.3)  100 (4.2)  226 (9.4) | 14 891 (56.1)  8 118 (30.6)  1 274 (4.8)  2 273 (8.6) | <0.001 |
| Reoperation for recurrence  Yes  No | 107 (4.5)  2 286 (95.5) | 681 (2.6)  25 875 (97.4) | <0.001 |

B-GHR, bilateral groin hernia repair. U-GHR, unilateral groin hernia repair. IPQ, Inguinal pain questionnaire.

Nominal and ordinal characteristics are reported as frequency. Percentages are presented in parentheses unless indicated otherwise.

^a)^ The ASA physical fitness grade refers to the Physical status classification system adopted in 1963 by the American Society of Anesthesiologists. Grade I–II refer to persons who are heathy or suffering from a mild systemic disease, and grade III–IV refer to persons who are suffering from severe systemic diseases some of which are life threatening.

^b)^ Combined hernias are defined as lateral hernia in combination with either cord lipoma or medial hernia. Any combination with femoral hernia is defined primarily as femoral hernias in the register.

^c)^ A Chi-square crosstabulation performed for each categorical variable in the bilateral and unilateral cohort with given p-values.

**Table S2.** Unadjusted analysis for the risk of chronic pain, patient dissatisfaction and reoperation for recurrence for the included study population.

| Outcomes for total repairs among responders | U-GHR | B-GHR |
| --- | --- | --- |
| Chronic pain | 1.0 | OR 1.06 (95% CI 0.99-1.15) |
| Patient dissatisfaction | 1.0 | OR 1.46 (95% CI 1.31-1.63) |
| Reoperation for recurrence | 1.0 | HR 1.63 (95% CI 1.41,1.89) |

OR, Odds Ratio; HR, hazard ratio; CI, Confidence Interval.

**Supplementary Appendixes**

STROBE Statement - checklist of items that should be included in reports of observational studies

|  | | **Item No.** | **Recommendation** | **Page  No.** | **Relevant text from manuscript** |
| --- | --- | --- | --- | --- | --- |
| **Title and abstract** | 1 | (*a*) Indicate the study’s design with a commonly used term in the title or the abstract | **1** | **Title: “Increased Chronic pain and Patient Dissatisfaction after simultaneous Bilateral groin hernia repair: prospective nationwide cohort study”** |  |
|  |  | (*b*) Provide in the abstract an informative and balanced summary of what was done and what was found | **3** | **ABSTRACT** |  |
| **Introduction** | | | | |  |
| Background/rationale | | 2 | Explain the scientific background and rationale for the investigation being reported | **4** | **INTRODUCTION** |
| Objectives | | 3 | State specific objectives, including any prespecified hypotheses | **4-5** | **INTRODUCTION** |
| **Methods** | | | | |  |
| Study design | | 4 | Present key elements of study design early in the paper | **6** | **METHODS: *Study design*** |
| Setting | | 5 | Describe the setting, locations, and relevant dates, including periods of recruitment, exposure, follow-up, and data collection | **6-8** | **METHODS: *Study population,***  ***PROM questionnaire, Bilateral repair, Study objectives, Statistical analysis.*** |
| Participants | | 6 | (*a*) *Cohort study*—Give the eligibility criteria, and the sources and methods of selection of participants. Describe methods of follow-up  *Case-control study*—Give the eligibility criteria, and the sources and methods of case ascertainment and control selection. Give the rationale for the choice of cases and controls  *Cross-sectional study*—Give the eligibility criteria, and the sources and methods of selection of participants | **6-8** | **METHODS: *Study population,***  ***PROM questionnaire, Bilateral repair, Study objectives, Statistical analysis.*** |
|  |  |  | (*b*) *Cohort study*—For matched studies, give matching criteria and number of exposed and unexposed  *Case-control study*—For matched studies, give matching criteria and the number of controls per case |  |  |
| Variables | | 7 | Clearly define all outcomes, exposures, predictors, potential confounders, and effect modifiers. Give diagnostic criteria, if applicable | **6-8** | **METHODS: *Study objectives, Statistical analysis.*** |
| Data sources/ measurement | | 8* | For each variable of interest, give sources of data and details of methods of assessment (measurement). Describe comparability of assessment methods if there is more than one group | **6-8** | **METHODS: *Study population,***  ***PROM questionnaire, Bilateral repair, Study objectives, Statistical analysis.*** |
| Bias | | 9 | Describe any efforts to address potential sources of bias | **8** | **METHODS: *Statistical analysis.*** |
| Study size | | 10 | Explain how the study size was arrived at | **6-8** | **METHODS: *Study population,***  ***PROM questionnaire, Bilateral repair, Study objectives, Statistical analysis.*** |

Continued on next page

| Quantitative variables | 11 | Explain how quantitative variables were handled in the analyses. If applicable, describe which groupings were chosen and why | **8** | **METHODS: *Statistical analysis.*** |
| --- | --- | --- | --- | --- |
| Statistical methods | 12 | (*a*) Describe all statistical methods, including those used to control for confounding | **8** | **METHODS: *Statistical analysis.*** |
|  |  | (*b*) Describe any methods used to examine subgroups and interactions | **8** | **METHODS: *Statistical analysis.*** |
|  |  | (*c*) Explain how missing data were addressed |  |  |
|  |  | (*d*) *Cohort study*—If applicable, explain how loss to follow-up was addressed  *Case-control study*—If applicable, explain how matching of cases and controls was addressed  *Cross-sectional study*—If applicable, describe analytical methods taking account of sampling strategy |  |  |
|  |  | (*e*) Describe any sensitivity analyses | **8** | **METHODS: *Statistical analysis.*** |
| **Results** | | | | |
| Participants | 13* | (a) Report numbers of individuals at each stage of study—eg numbers potentially eligible, examined for eligibility, confirmed eligible, included in the study, completing follow-up, and analysed | **9-10**  **Figure 1** | **RESULTS**  **Flowchart** |
|  |  | (b) Give reasons for non-participation at each stage | **6**  **Figure 1** | **Methods: *Study design.***  **Flowchart** |
|  |  | (c) Consider use of a flow diagram | **Figure 1** | **Flowchart** |
| Descriptive data | 14* | (a) Give characteristics of study participants (eg demographic, clinical, social) and information on exposures and potential confounders | **9-10** | **RESULTS: *Patient characteristics***  ***Non-responders.*** |
|  |  | (b) Indicate number of participants with missing data for each variable of interest | **Table 1**  **Table 2**  **Table 3** | ***Table 1***  ***Table 2***  ***Table 3*** |
|  |  | (c) *Cohort study*—Summarise follow-up time (eg, average and total amount) | **Table 1**  **Table 2**  **Table 3** | ***Table 1***  ***Table 2***  ***Table 3*** |
| Outcome data | 15* | *Cohort study*—Report numbers of outcome events or summary measures over time | **9-10** | **RESULTS: *Chronic pain, Patient dissatisfaction, Reoperation for recurrence, Risk factors for outcomes exclusively after B-GHR, Non-responders.*** |
|  |  | *Case-control study—*Report numbers in each exposure category, or summary measures of exposure |  |  |
|  |  | *Cross-sectional study—*Report numbers of outcome events or summary measures |  |  |
| Main results | 16 | (*a*) Give unadjusted estimates and, if applicable, confounder-adjusted estimates and their precision (eg, 95% confidence interval). Make clear which confounders were adjusted for and why they were included | **9-10** | **RESULTS: *Patient characteristics Chronic pain, Patient dissatisfaction, Reoperation for recurrence, Risk factors for outcomes exclusively after B-GHR, Non-responders.*** |
|  |  | (*b*) Report category boundaries when continuous variables were categorized |  |  |
|  |  | (*c*) If relevant, consider translating estimates of relative risk into absolute risk for a meaningful time period |  |  |

Continued on next page

| Other analyses | 17 | Report other analyses done—eg analyses of subgroups and interactions, and sensitivity analyses | **9-10** | **RESULTS: *Patient characteristics Chronic pain, Patient dissatisfaction, Reoperation for recurrence, Risk factors for outcomes exclusively after B-GHR, Non-responders.*** |
| --- | --- | --- | --- | --- |
| **Discussion** | | | | |
| Key results | 18 | Summarise key results with reference to study objectives | **10** | **DISCUSSION** |
| Limitations | 19 | Discuss limitations of the study, taking into account sources of potential bias or imprecision. Discuss both direction and magnitude of any potential bias | **13** | **DISCUSSION** |
| Interpretation | 20 | Give a cautious overall interpretation of results considering objectives, limitations, multiplicity of analyses, results from similar studies, and other relevant evidence | **10-14** | **DISCUSSION** |
| Generalisability | 21 | Discuss the generalisability (external validity) of the study results | **12** | **DISCUSSION** |
| **Other information** | |  | | |
| Funding | 22 | Give the source of funding and the role of the funders for the present study and, if applicable, for the original study on which the present article is based | **1** | **Title page: *Source of funding*** |

*Give information separately for cases and controls in case-control studies and, if applicable, for exposed and unexposed groups in cohort and cross-sectional studies.

**Note:** An Explanation and Elaboration article discusses each checklist item and gives methodological background and published examples of transparent reporting. The STROBE checklist is best used in conjunction with this article (freely available on the Web sites of PLoS Medicine at http://www.plosmedicine.org/, Annals of Internal Medicine at http://www.annals.org/, and Epidemiology at http://www.epidem.com/). Information on the STROBE Initiative is available at www.strobe-statement.org.
